# Preclinical and Clinical Evidence for a Distinct Regulation of Mu Opioid and Type 1 Cannabinoid Receptor Genes Expression in Obesity

**DOI:** 10.3389/fgene.2019.00523

**Published:** 2019-06-14

**Authors:** Mariangela Pucci, Maria Vittoria Micioni Di Bonaventura, Valeria Vezzoli, Elizabeta Zaplatic, Marcella Massimini, Stefania Mai, Alessandro Sartorio, Massimo Scacchi, Luca Persani, Mauro Maccarrone, Carlo Cifani, Claudio D’Addario

**Affiliations:** ^1^Faculty of Bioscience and Technology for Food, Agriculture and Environment, University of Teramo, Teramo, Italy; ^2^Pharmacology Unit, School of Pharmacy, University of Camerino, Camerino, Italy; ^3^Lab of Endocrine and Metabolic Research, Istituto Auxologico Italiano IRCCS, Milan, Italy; ^4^Department of Clinical Sciences and Community Health, University of Milan, Milan, Italy; ^5^Department of Medicine, Campus Bio-Medico University of Rome, Rome, Italy; ^6^Department of Clinical Neuroscience, Karolinska Institutet, Stockholm, Sweden

**Keywords:** obesity, endocannabinoid system, opioid system, DNA methylation, biomarker

## Abstract

Among endogenous signaling networks involved in both rewarding and homeostatic mechanisms of obesity, a relevant role is played by the endocannabinoid (ECS) and the opioid (EOS) systems. We here studied the transcriptional regulation of ECS and EOS genes in the hypothalamus of Diet-induced obesity rats, a preclinical model of obesity, as well as in humans with obesity and healthy controls. A significant and selective increase in type 1 cannabinoid receptor gene (*Cnr1*) expression was observed at the beginning of obesity development (5 weeks on high fat diet) as well as after 21 weeks of high diet exposure. After 5 weeks on high fat diet, selective up-regulation of mu opioid receptor gene (*Oprm1*) expression was also observed. Consistently, epigenetic studies showed a selective and significant decrease in DNA methylation at specific CpG sites at both gene promoters in overweight rats, but only after 5 weeks on high fat diet. Moreover, significantly lower levels of DNA methylation were observed at selected CpG sites of both receptor gene promoters, analyzed in peripheral blood mononuclear cells from younger (<30 years old) humans with obesity, as well as in those with shorter time length from disease onset. Taken together, we here provide evidence of selective, synergistic and time-dependent transcriptional regulation of *CNR1* and *OPRM1* genes in overweight rats, as well as in human subjects. These alterations in genes regulation could contribute to the development of the obese phenotype, and we thus suggest *CNR1* and *OPRM1* epigenetic modulation as possible biomarkers of obesity development. Due to the reversible nature of the epigenetic hallmark, our data might also open new avenue to early environmental strategies of intervention.

## Introduction

Obesity is a growing public health threat, potentially affecting emotional, and physical health with a relevant mortality rate and a high burden of disease for Western societies. Core symptoms of this disease are disturbance of eating habits and inability to control body weight, thus leading to an imbalance between energy intake and expenditure. In recent years several approaches have been used to treat obesity, yet the efficacy of the few medications proven useful remains a matter of debate. Thus, effective and safe treatments are urgently needed (Patel and Stanford, 2018). Efforts should be made to disclose the causes that make individuals more vulnerable to the development of obesity, in order to gain useful information for alleviating or even preventing occurrence of this condition. Genetic predisposition has been implicated in disease susceptibility (Stice, 2002; Striegel-Moore and Bulik, 2007), however, differences in phenotype heterogeneity point also to the relevant role of the environment and lifestyle (Hill et al., 2003). Molecular research has formed the basis to understand how environmental factors (i.e., dieting) may facilitate disease progression by engaging epigenetic mechanisms (van Dijk et al., 2015), helping also to understand the increasing epidemic of the disease in the last years within the same genome. It is in fact well known that the latter mechanisms can evoke transient changes (although how long they actually last is unclear) in gene expression, involving chemical modifications of DNA that do not affect the actual DNA sequence of the organism. The understanding of how transcriptional regulation might affect individual risk of developing obesity represents a major challenge in research and may provide invaluable help for the development of preventive strategies, or of more effective therapeutics. Central regulation of food intake is rather complex, and thus different endogenous key players should be considered. For instance, it is clear that rewarding properties of food, possibly leading to drug-like food addiction (D’Addario et al., 2014; Mancino et al., 2015), are responsible for disease development and share neuronal pathways that seem to overlap with drug addiction (Volkow and Wise, 2005). These neural circuitries, driving eating behaviors in the brain to ensure food-intake and to regulate caloric balance, are thus controlled not only by homeostatic mechanisms but also by reward systems to promote motivational, hedonically driven feeding (Coccurello and Maccarrone, 2018). Among the endogenous systems involved in both rewarding and homeostatic mechanisms, a relevant role is played by both the endocannabinoid (ECS) and the opioid (EOS) systems (Cota et al., 2003a; Tanda and Goldberg, 2003), which functionally interact with each other in mediating neurological functions (Manzanares et al., 1999; Tanda and Goldberg, 2003). In feeding regulation, both ECS and EOS seem to contribute to the reward aspect of eating (Saper et al., 2002; Yeomans and Gray, 2002; Cota et al., 2003b). Of note, several preclinical studies reported that both opioid and cannabinoid receptor agonists stimulate food intake (Reid, 1985; Foltin et al., 1988; Williams et al., 1998; Glass et al., 1999; Williams and Kirkham, 1999), that is instead reduced by antagonists (Recant et al., 1980; Marks-Kaufman et al., 1984; Colombo et al., 1998; Di Marzo et al., 2001; Glass et al., 2002; Hildebrandt et al., 2003; Ravinet Trillou et al., 2003; Statnick et al., 2003; Vickers et al., 2003) with suppression of body weight gain in rodents. It has been also observed in human trials that opioid antagonists (naloxone, naltrexone, or nalmefene) may be helpful in the short term to suppress appetite, even though caution should be taken for possible long term use because of side effects and limited weight loss (Nathan and Bullmore, 2009). In this context, it should be recalled that rimonabant, a selective type 1 cannabinoid receptor (CB_1_) antagonist able to reduce body weight (Rinaldi-Carmona et al., 1995; Di Marzo and Despres, 2009), was an approved drug on the European market, but was withdrawn because of an increased risk of psychiatric disorders (Johansson et al., 2009).

Moreover, combination therapies [i.e., *Contrave* (Apovian et al., 2013) and *Qysmia* (Gadde et al., 2011)] also held promise for obesity treatment, and preclinical studies performed using a combination of opioid and cannabinoid antagonists (naloxone and nor-BNI with rimonabant) demonstrated enhanced feeding reduction when compared to that evoked by rimonabant alone (Lockie et al., 2011, 2015).

Against this background, the aim of this study was to advance our knowledge about the molecular mechanisms engaging ECS and EOS in the development of obesity, in order to identify disease biomarkers and disclose new molecular clues to be targeted by innovative strategies of pharmacological intervention. By using a well-established rat model of Diet-Induced Obesity (DIO), we first investigated regulation of ECS and EOS genes expression during disease progression in the hypothalamus, a brain region involved in appetite regulation (Bazhan and Zelena, 2013), where the role of ECS (Berthoud, 2012; Zeltser et al., 2012; Ramírez-López et al., 2015) and EOC (Dum et al., 1983; Nogueiras et al., 2012; Romero-Picó et al., 2018) in feeding response modulation has been deeply investigated and already reported. We followed gene expression regulation at the beginning of obesity development (i.e., after 5 weeks on high fat diet), as well as when the phenotype was well-established (i.e., after 21 weeks on high fat diet). Among the epigenetic mechanisms possibly responsible for altered gene expression, we focused on DNA methylation, that consists of a methyl group transfer to the position 5 of the cytosine pyrimidine ring in a cytosine guanine dinucleotide (CpG), which ultimately blocks the binding of transcription factors thus causing chromatin compaction and gene silencing (Zhu et al., 2016). Of note, by using the same DIO animal model considered here, we already showed the epigenetic regulation of other obesity genes in the progression of the disorder (Cifani et al., 2015).

In addition, in this work, we also analyzed the DNA methylation status of key ECS and EOS genes in peripheral blood mononuclear cells (PBMCs) from a subset of humans with obesity. It is important to recall that PBMCs contain the complete epigenetic machinery present in neurons (Arosio et al., 2014), and are considered a convenient substitute for cerebral markers (Woelk et al., 2011) that are readily accessible and reflects the molecular processes occurring in the central nervous system (Gladkevich et al., 2004). PBMCs have been already proposed in obesity studies as a valuable tool to monitor metabolic recovery in weight loss strategies (Reynés et al., 2015), and thus their study in this disorder is of clear relevance to detect altered pathways (metabolic or signaling) that could be transferred to the brain. Moreover, it has been well-documented since many years the activation of the ECS in human obese subjects and the regulation of EOS components in obesity and related eating disorders in the periphery (Jimerson and Wolfe, 2004).

## Materials and Methods

### Animals and Diet Composition

**FIGURE 1:**
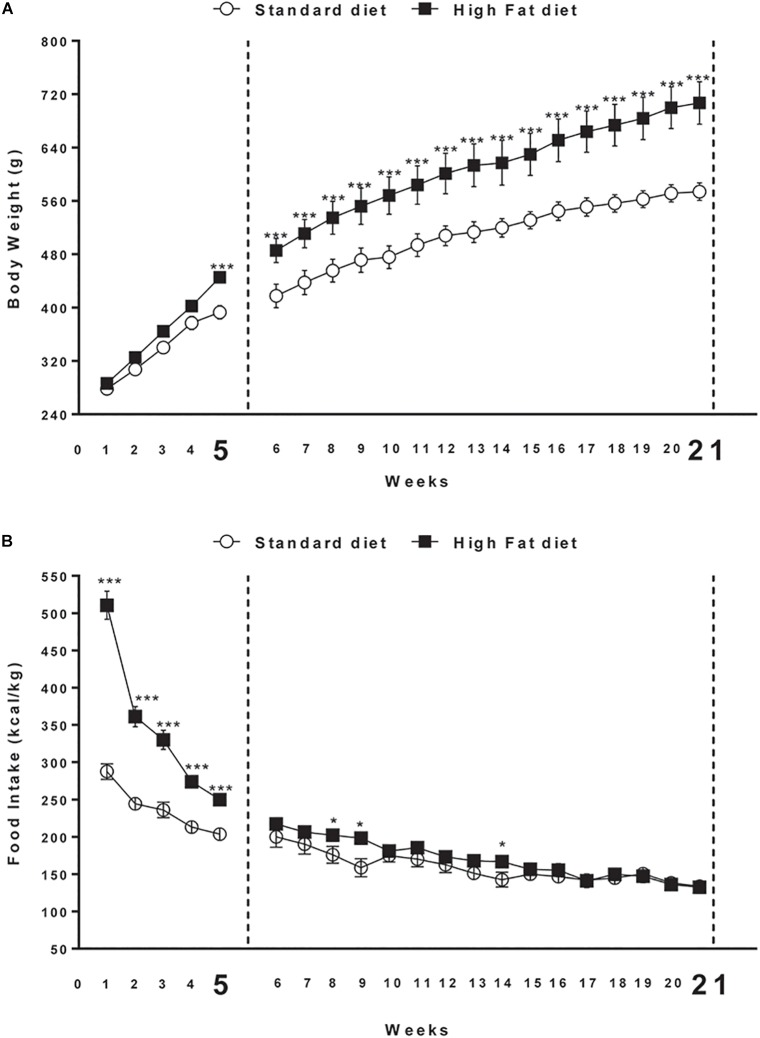
**(A)** Body weight and **(B)** cumulative food intake (kcal) measured weekly in rats exposed for 5 and 21 weeks to high fat (HFD) or standard diet (STD). Significant differences are indicated: ^∗∗∗^*P* < 0.001, ^∗^*P* < 0.05 vs. STD.

Male Sprague Dawley rats (Charles River; total *n* = 38; 225–250 g, 7 weeks old at the beginning of the experiments) were used. Rats were housed in individual cage sunder 12:12 h light/dark cycle (lights on at 9:00 a.m.) with access to food and water *ad libitum* for 2 weeks before the experiments. They were kept in a room at constant temperature (20–22°C) and humidity (45–55%). All procedures involving rats were carried out in accordance with the Institutional Guidelines and complied with the Italian Ministry of Health and associated guidelines from European Communities Council Directive. Rats were randomly divided into two groups with comparable mean body weight (no significant difference). The first group (*n* = 16) was the control group and was fed with standard laboratory chow *ad libitum* (4RF18, Mucedola, Settimo Milanese, Italy; 2.6 kcal/g); the second group (*n* = 22), was fed with high energy diet (45% fat, 35% Carbohydrate, 20% Protein) *ad libitum* (D12451, Research Diets, Inc., New Brunswick, NJ, United States; 5.24 kcal/g). After 5 weeks, 6 of the 22 rats fed with high fat diet did not significantly increase body weight in comparison to rats fed with chow. These resistant rats were excluded from the study (Cifani et al., 2015) because they did not develop obese phenotype. At the end of the 5 weeks, eight Chow and eight DIO rats were sacrificed by decapitation. The remaining animals (additional eight Chow and eight DIO rats) were maintained on their respective diets for 21 weeks, and then were sacrificed. Brains were quickly removed and the whole hypothalamus was manually dissected (from Bregma level -0.26 to Bregma level -4.20) (Paxinos and Watson, 1998). The tissues were collected and stored at -80°C until further analysis. Body weight and food intake were daily recorded (Figure 1).

### Human Subjects

#### Patients and Methods

##### Editorial policies and ethical considerations

The study, accomplishing the Declaration of Helsinki, was approved by the Ethic Committee of IRCCS – Istituto Auxologico Italiano (RBFR12DELS_004/05C302_2013), and all patients or their tutors gave a written informed consent.

##### Study population

The entire cohort consists of 63 humans with obesity recruited from the San Giuseppe Hospital’s and San Luca Hospital’s (Istituto Auxologico Italiano) since 2012. Clinical characteristics of our cohort are reported in Table 1. Anonymous patient data, referred to the time of diagnosis, were collected either prospectively or retrospectively and a clinical database was created. We performed the same epigenetic analysis in 58 non-obese blood donors matching for age and sex (Males: No. = 32, age = 42.27 ± 2.97; Females: No. = 26, age 40.87 ± 4.12).

**Table 1:** Clinical characteristics and biochemical parameters of subjects enrolled for the DNA methylation study.

Obese subjects	Males	Females
No.	27	36
Age, years ± SD	44.15 ± 4.45	41.62 ± 4.135
BMI, kg/m^2^ ± SD	40.69 ± 1.52	40.23 ± 1.17
Glicemia ± SD	115.30 ± 9.18	98.89 ± 4.29
Cholesterol-total, mg/dL ± SD	172.70 ± 8.74	175.20 ± 7.24
Cholesterol-HDL, mg/dL ± SD	93.85 ± 9.93	104.40 ± 11.98
Cholesterol-LDL, mg/dL ± SD	60.70 ± 6.46	65.47 ± 4.67
Triglycerides, mg/dL ± SD	135.00 ± 11.11	116.80 ± 7.90
Systolic blood pressure, mmHg ± SD	125.20 ± 1.88	123.10 ± 1.25
Diastolic blood pressure, mmHg ± SD	77.22 ± 1.11	78.06 ± 1.34


##### Data collection

In this study all humans with obesity had physical examination and biochemistry analyses. To perform the body weight and height measurements, subjects were dressed in light clothing without socks and shoes. Weight was measured (in kg to two decimal places) with a digital balance scale. Height was measured with a wall-mounted stadiometer (to the nearest millimeter) using the stretch technique. Body mass index (BMI) was calculated using the Quetelet’s formula as weight (in kg) divided by height (in m^2^). Systolic (SBP) and diastolic (DBP) blood pressure were measured in the seated position with a Tema Certus sphygmomanometer and an appropriately sized cuff on the right arm. These values were obtained as the average of three measurements. Blood samples were separated by centrifugation after clotting, and aliquots of serum or plasma supernatants were processed for routine measurements and stored at -80°C until assay. Routine laboratory data included levels of glucose, total cho- lesterol, high-density (HDL) and low-density lipoprotein (LDL) cholesterol, triglycerides (TG) determined by enzymatic assays (Roche Diagnostics GmbH, Mannheim, Germany). Anthropometrical and biochemical measurements were made once for each participant. According to the World Health Organization guidelines, obesity was defined as BMI ≥ 30 kg/m^2^ (WHO, 2000).

Non-obese blood donors were selected in accordance with National mandatory standards for blood donor selection. They were known to be free from chronic disease (including diabetes, hypertension, dyslipidemia, and cardiovascular disease), not taking any drugs and with BMI < 29.9 kg/m^2^.

### Molecular Biology Studies

#### Real-Time Quantitative PCR

Total RNA was isolated using TRIzol reagent (Thermo Fisher Scientific) according to the specifications of the instructions, from rat dissected Hypothalamus and from PBMCs, separated by density gradient using the Lympholyte-H kit (Cedarlane Laboratories, Canada). Each 0.5 μg of total RNA was reverse-transcribed into complementary DNA using a RevertAid RT Reverse Transcription Kit (Thermo Scientific). Random hexamers and oligo-dT primers were used in the RT reaction in an unbiased manner Quantitative PCR was performed using an Applied Biosystems 7500 Real-time PCR system (Thermo Fisher Scientific) with SensiFAST SYBR Low-ROX kit (Bioline) in a total reaction volume of 10 μl according to the manufacturer’s instructions. The thermal cycles were as follows: initial denaturation at 95°C for 2 min; 45 cycles of denaturation at 95°C for 15 s; annealing and extension at 60°C for 15 s. Relative mRNA expression levels were calculated using the 2^-DDCt^ method and normalized to two internal control, β-actin and GAPDH. The primers used for the amplification of ECS and EOS genes are reported in Supplementary Table S1.

#### DNA Methylation Analysis by Pyrosequencing

**FIGURE 2:**
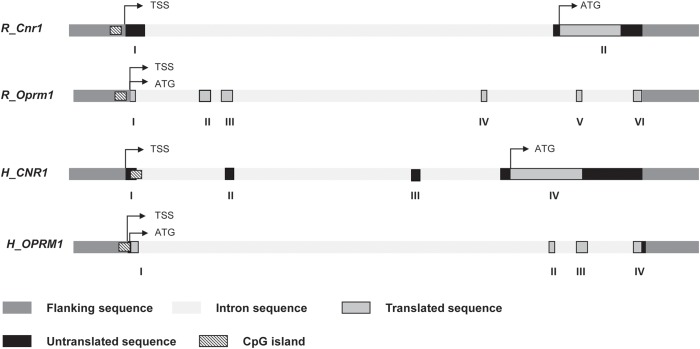
Schematic representation of rat and human *Cnr1* and *Oprm1* genes. Position of transcription start site (TSS), translation start code (ATG), exons and introns, CpG island are depicted. Details of the sequences under study for DNA methylation are shown in Supplementary Table S2.

Genomic DNA was extracted from hypothalamic region and PBMC by using TRIzol Reagent (Life Technologies) with the concentration and purity detected by NanoDrop spectrophotometer (NanoDrop Technologies, United States). DNA was subjected to bisulfite modification by means of a commercially available modification kit (Zymo Research). Pyrosequencing was used to quantify the methylation levels of individual CpG sites. The sequencing was performed for all the study samples on a PyroMark Q24 ID using Pyro Mark Gold reagents (Qiagen). Primers for rat and human *CNR1*, the gene coding for the cannabinoid receptor type 1 (CB_1_) (targeting in rats eight CpG sites and in humans five CpGs) and *OPRM1*, the gene coding for mu opioid receptor (MOP) (targeting in rats four CpG sites and in humans five CpGs), were generated according to Pyro Mark Assay Design software version 2.0 (Qiagen). The schematic representation of CpG island at *CNR1* and *OPRM1* promoter regions and the details of the pyrosequencing assay are illustrated in Figure 2 and Supplementary Table S2. Bisulfite treated DNA was amplified by PyroMark PCR Kit (Qiagen) according to the manufacturer’s protocol. Polymerase chain reaction conditions were as follows: 95°C for 15 min, followed by 45 cycles of 94°C for 30 s, 56°C for 30 s, 72°C for 30 s, and finally, 72°C for 10 min. Polymerase chain reaction products were verified by agarose electrophoresis. Pyromark Q24 ID version 1.0.9 software, which generates and automatically analyzes the resulting pyrograms, was used to calculate the methylation percentage mC/(mC + C) (where mC is methylated cytosine and C is unmethylated cytosine), for each CpG site, allowing quantitative comparisons. Quantitative methylation results were considered both as a percentage of individual CpG sites and as an average of the methylation percentage of the all the investigated CpGs.

#### Statistical Analysis

All results are expressed as mean ± SEM. Statistical differences of genes expression and DNA methylation changes at gene promoters in both human and animal samples were determined using Prism version 6 (Graph-Pad Software, San Diego, CA, United States). In behavioral experiments, data were analyzed by two-way ANOVA with the animal group as the between-subject variable and time as the within-subject variable followed by *post hoc* comparison carried out by the Bonferroni test. In molecular biology studies, data were analyzed by two-way ANOVA with the animal group as the between-subject variable and CpG sites as the within-subject variable. Significant differences induced by diet were analyzed using the Mann–Whitney *U* test. DNA methylation at each CpG site was analyzed using the Mann–Whitney test and Sidak–Bonferroni correction was used for the multiple comparisons. The *P*-values < 0.05 were considered to be statistically significant.

## Results

### High Fat Diet Effect on Body Weight and Food Intake

At the beginning of the study, body weight of rats in the high fat diet group (278.3 ± 7.0 g) did not differ significantly from that of the rats in the control group (286.2 ± 6.3 g) [*F*_(1,30)_ = 0.7; *P* > 0.05]. At the end of the fifth week, two-way ANOVA showed a significant difference in body weight between the groups (Diet: [*F*_(1,30)_ = 7.2; *P* < 0.05]; Time: [*F*_(4,120)_ = 297.5; *P* < 0.01]; Interaction: [*F*_(4,120)_ = 6.7; *P* < 0.01]). At the fifth week time point, *post hoc* test showed that body weight of DIO rats began to be significantly higher compared to that of control group (*P* < 0.05).

At the end of the 21st week, two-way ANOVA showed a significant difference in body weight between the groups (Diet: [*F*_(1,14)_ = 10.0; *P* < 0.01]; Time: [*F*_(20,280)_ = 230.2; *P* < 0.001]; Interaction: [*F*_(20,280)_ = 5.3; *P* < 0.001]). At the same time point (21st week) of free access to high fat diet, *post hoc* test showed that body weight of DIO rats was significantly higher in comparison to body weight of rats fed with standard diet (591.0 ± 2.2 g) (*P* < 0.001; Figure 1A).

Overall ANOVA showed a significant difference in energy intake (kcal/kg) between the groups in the first 5 weeks (Diet: [*F*_(1,30)_ = 110.9; *P* < 0.001]; Time: [*F*_(4,120)_ = 117.7; *P* < 0.001]; Interaction: [*F*_(4,120)_ = 29.8; *P* < 0.001]). At the end of the 21st week, two-way ANOVA showed a significant interaction between diet and time [*F*_(4,120)_ = 29.9; *P* < 0.001]. Significant differences at each time point are shown in Figure 1B. Open field test showed significant difference between the groups in Distance travel, Vertical count, Grooming and Zone Entries only after 21 weeks of diet exposure (Supplementary Table S3).

### Regulation of ECS and EOS Genes Transcription in the Hypothalamus

#### ECS

**FIGURE 3:**
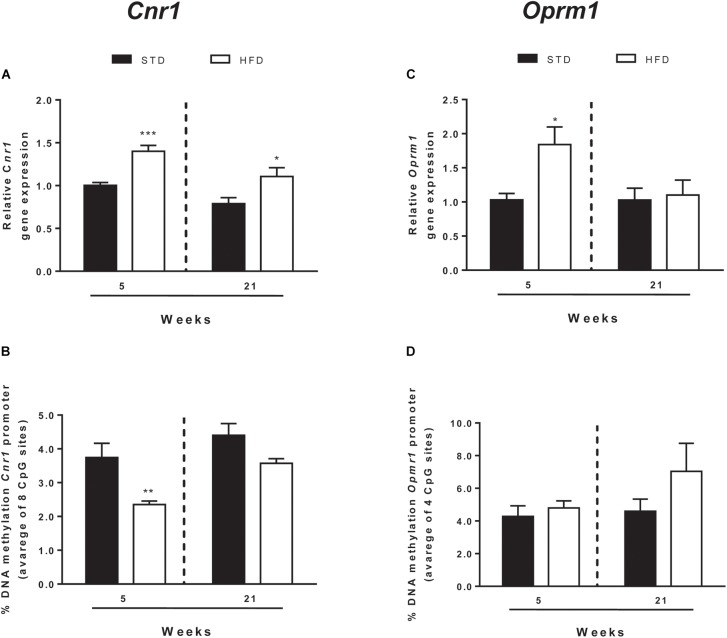
*Cnr1* and *Oprm1* transcriptional regulation. *Cnr1* relative gene expression **(A)** and DNA methylation at gene promoter **(B)** and *Oprm1* relative gene expression **(C)**, and DNA methylation at gene promoter **(D)** analyzed in the hypothalamus of rats exposed for 5 and 21 HFD and/or STD Gene expression data are reported as 2^-DDCt^ values calculated by Delta–Delta Ct (DDCt) method vs. STD (5 weeks) posed equal to 1. DNA methylation data are presented as the mean of the methylation % values of individual CpG sites under study as well as of the average (Ave) of the eight CpG sites ± SEM. ^∗∗∗^*P* < 0.001, ^∗∗^*P* < 0.01, ^∗^*P* < 0.05 vs. STD.

In addition to CB_1_, endocannabinoid signaling is based on a rather complex array of proteins that form the so-called ECS. These include additional receptor targets, like type-2 cannabinoid receptor (CB_2_) and transient receptor potential vanilloid-1 (TRPV1) channels, and metabolic enzymes involved in biosynthesis and degradation of endocannabinoids (Chiurchiù et al., 2018). All these elements of ECS were analyzed by qRT-PCR (Figure 3 and Table 2).

**Table 2:** Gene expression of ECS and EOS elements in the hypothalamus of normal weight and overweight rats exposed for 5 and 21 weeks to high fat and/or standard diet, reported as 2^-DDCt^ values calculated by Delta–Delta Ct (DDCt) method vs. normal weight (5 weeks) posed equal to 1.

HYP		5 weeks		21 weeks	
Gene		STD	HFD	STD	HFD
ECS	*Cnr1*	1.03 ± 0.03	1.42 ± 0.09^a^	0.79 ± 0.07	1.18 ± 0.16^c^
	*Cnr2*	1.21 ± 0.25	1.16 ± 0.70	1.98 ± 0.57	1.90 ± 0.59
	*Gpr55*	1.22 ± 0.29	1.10 ± 0.21	1.44 ± 0.14	1.54 ± 0.27
	*Trpv1*	1.24 ± 0.30	1.34 ± 0.45	1.22 ± 0.29	0.78 ± 0.06
	*Nape-Pld*	1.06 ± 0.10	1.08 ± 0.15	1.10 ± 0.13	1.08 ± 0.18
	*Faah*	1.09 ± 0.17	0.87 ± 0.12	1.36 ± 0.12	1.48 ± 0.10
	*Dagl*	1.05 ± 0.13	1.49 ± 0.26	0.74 ± 0.07	0.58 ± 0.05
	*Magl*	1.04 ± 0.11	1.14 ± 0.14	0.94 ± 0.08	0.88 ± 0.13
Opiod system	*Oprl1*	0.98 ± 0.05	1.23 ± 0.08	1.11 ± 0.12	1.15 ± 0.02
	*Oprm1*	1.03 ± 0.09	1.84 ± 0.26^b^	1.03 ± 0.17	1.10 ± 0.22
	*Oprd1*	1.03 ± 0.11	1.36 ± 0.22	0.73 ± 0.09	0.71 ± 0.07
	*Oprk1*	1.05 ± 0.17	1.23 ± 0.30	1.29 ± 0.23	1.19 ± 0.30
	*Pomc*	1.18 ± 0.33	0.89 ± 0.22	1.19 ± 0.30	0.73 ± 1.19
	*Pnoc*	1.03 ± 0.10	1.48 ± 0.17	1.00 ± 0.13	0.89 ± 0.11
	*Pdyn*	1.04 ± 0.11	1.30 ± 0.23	1.02 ± 0.12	0.97 ± 0.08
	*Penk*	1.01 ± 0.04	0.99 ± 0.15	1.06 ± 0.13	1.15 ± 0.17


*^a^p < 0.05 vs. STD; ^*b*^*p* < 0.01 vs. STD; ^*c*^*p* < 0.001 vs. STD.*

After 5 and 21 weeks, statistical analysis showed no significant changes of ECS elements, except for *Cnr1*, the gene encoding for CB_1_ (Table 2). Two-way ANOVA showed that mRNA levels were affected by time [*F*_(1,25)_ = 10.50, *P* = 0.003] and diet [*F*_(1,25)_ = 20.72, *P* = 0.0001], without a significant interaction between these two factors [*F*_(1,25)_ = 0.27, *P* = 0.607]. *T*-test revealed a selective and significant increase in *Cnr1* mRNA levels in HFD rats with respect to STD animals at both time points analyzed (5 weeks = *P* < 0.0006; 21 weeks = *P* < 0.036; Figure 3A).

DNA methylation analysis of each CpG site, and as an average of all eight CpG sites analyzed at the *Cnr1* promoter, showed significant changes in rats after 5 weeks on high fat diet (*t*-test: *P* = < 0.006; Figure 3B and Table 3). Two-way ANOVA showed that DNA methylation was affected by time [*F*_(1,26)_ = 6.62, *P* = 0.016] and diet [*F*_(1,26)_ = 5.34, *P* = 0.029], without a significant interaction between these two factors [*F*_(1,26)_ = 0.024, *P* = 0.876].

**Table 3:** DNA methylation changes at *Cnr1* gene promoter in the hypothalamus of normal weight and overweight rats exposed for 5 weeks to high fat and/or standard diet.

*Cnr1*	5 weeks		21 weeks	
CpG sites	STD	HFD	STD	HFD
1	4.55 ± 0.48	4.24 ± 0.42	5.11 ± 0.31	5.28 ± 0.67
2	4.76 ± 0.64	2.30 ± 0.28^b^	5.49 ± 0.63	4.64 ± 0.29
3	3.37 ± 0.45	1.37 ± 0.10^c^	3.83 ± 0.35	2.77 ± 0.26
4	6.36 ± 0.87	3.81 ± 0.28^a^	8.17 ± 0.73	6.15 ± 028
5	1.31 ± 0.21	1.01 ± 0.20	1.69 ± 0.14	1.43 ± 0.18
6	2.22 ± 0.23	1.69 ± 0.13	2.35 ± 0.25	2.14 ± 0.20
7	3.13 ± 0.45	2.23 ± 0.28	3.64 ± 0.29	2.67 ± 0.28
8	3.77 ± 0.35	2.60 ± 0.25^a^	4.30 ± 0.48	3.21 ± 0.23
Average	3.72 ± 0.43	2.46 ± 0.11^a^	4.33 ± 0.32	3.54 ± 0.17


*^a^p < 0.05 vs. STD; ^*b*^*p* < 0.01 vs. STD; ^*c*^*p* < 0.001 vs. STD.*

**FIGURE 4:**
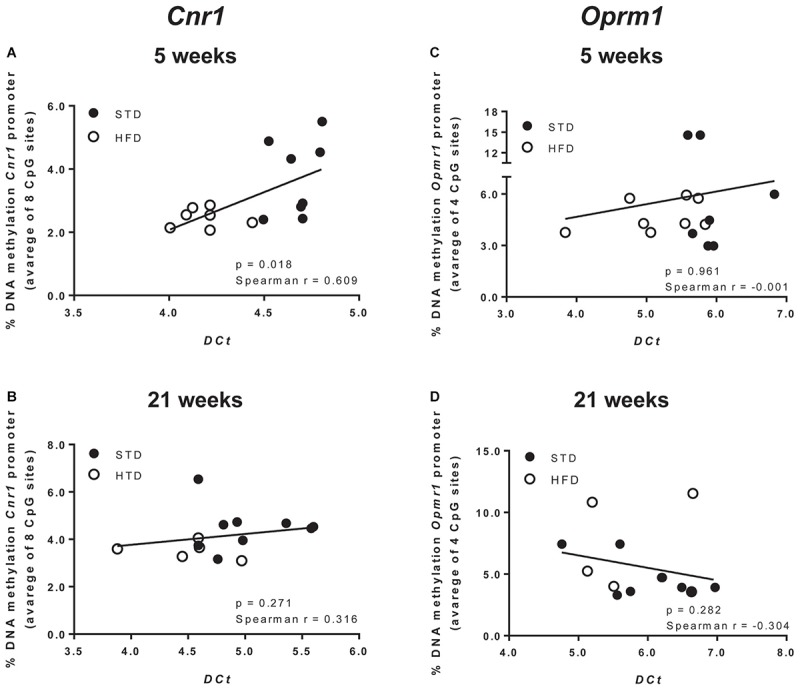
Correlation between Cnr1 expression and % change of DNA methylation after 5 **(A)** and 21 weeks of diet **(B)**. Correlation between *Oprm1* expression and % change of DNA methylation after 5 **(C)** and 21 weeks of diet **(D)**. Data were analyzed by Spearman’s rank correlation coefficient.

Moreover, a *t*-test corrected by Sidak–Bonferroni multiple comparisons indicated a significant decrease of *Cnr1* methylation levels at sites 2, 3, 4, and 8, as well as in the average of the eight sites analyzed, when compared to the STD group. No changes in DNA methylation were observed after 21 weeks (Table 3 and Figure 3B). Consistently, a correlation between gene expression and average DNA methylation was also observed in overweight and normal weight animals after 5 weeks of diet (Spearman *r* = 0.609; *P* = 0.018; Figure 4A), but not after 21 weeks (Spearman *r* = 0.316; *P* = 0.271; Figure 4B).

#### EOS

EOS consists of four receptors, mu (OPRM1), delta (OPRD1), kappa (OPRK1), and Opioid related nociceptin receptor 1 (OPRL1) which are activated by endogenous opioid peptides processed from four protein precursors, proopiomelanocortin (POMC), proenkephalin (PENK), prodynorphin (PDYN) and pronociceptin (PNOC) (Corbett et al., 2006; Bodnar, 2010). Among EOS elements, only the expression of genes encoding for the receptors *Oprm1* and *Oprd1* were altered (Figure 3 and Table 4). Two-way ANOVA showed that *Oprm1* mRNA levels were affected by diet [*F*_(1,25)_ = 4.90, *P* = 0.036] but not by time [*F*_(1,25)_ = 3.45, *P* = 0.07], and there was no significant interaction between these two factors [*F*_(1,25)_ = 3.43, *P* = 0.07]. *T*-test revealed a selective and significant increase in *Oprm1* mRNA levels in rats after 5 weeks on high fat diet with respect to STD animals (5 weeks = *P* < 0.05; Figure 3C and Table 4).

**Table 4:** DNA methylation changes at *Oprm1* gene promoter in the hypothalamus of normal weight and overweight rats exposed for 5 and 21 weeks to high fat and/or standard diet.

*Oprm1*	5 weeks		21 weeks	
CpG sites	STD	HFD	STD	HFD
1	3.56 ± 0.62	4.16 ± 0.40	3.05 ± 1.14	4.64 ± 1.00
2	7. 30 ± 1.13	8.55 ± 0.57	8. 05 ± 1.16	7.31 ± 0.52
3	2. 81 ± 0.45	2.92 ± 0.67	3. 38 ± 0.59	5.15 ± 1.40
4	3.46 ± 0.43	3.54 ± 0.34	3.90 ± 0.68	4.82 ± 1.12
Average	4.28 ± 0.64	4.80 ± 0.44	4.60 ± 0.75	7.04 ± 1.72


DNA methylation analysis of each CpG site, and as an average of all four CpG sites analyzed at the *Oprm1* promoter, did not show any significant change in rats exposed for 5 and 21 weeks to high fat diet (Table 4 and Figure 3D), nor any correlation between gene expression and average DNA methylation (at 5 weeks: Spearman *r* = -0.001; *P* = 0.961; at 21 weeks: Spearman *r* = -0.304; *P* = 0.282; Figure 4C,D).

### DNA Methylation at *CNR1* and *OPRM1* Promoter Regions in Clinical Samples

**FIGURE 5:**
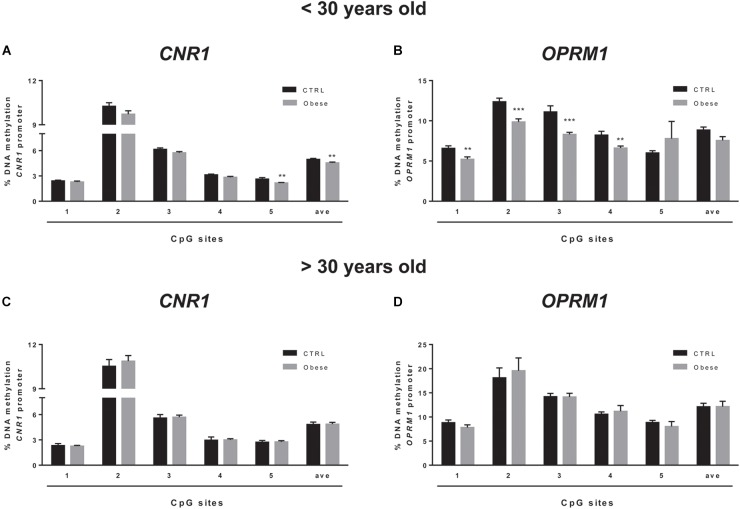
Comparison of DNA methylation status at human *CNR1* **(A,C)** and *OPRM1* **(B,D)** promoters in the obese population and control (CTRL) subjects stratified based on age (**A,B** = < 30 years old; **C,D** > 30 years old). The bars represent the mean of the % of methylation values of individual CpG sites under study as well as of the average (ave) of the CpG sites ± the SEM. Significant differences are indicated: ^∗∗^*P* < 0.01; ^∗∗∗^*P* < 0.001 vs. CTRL.

**FIGURE 6:**
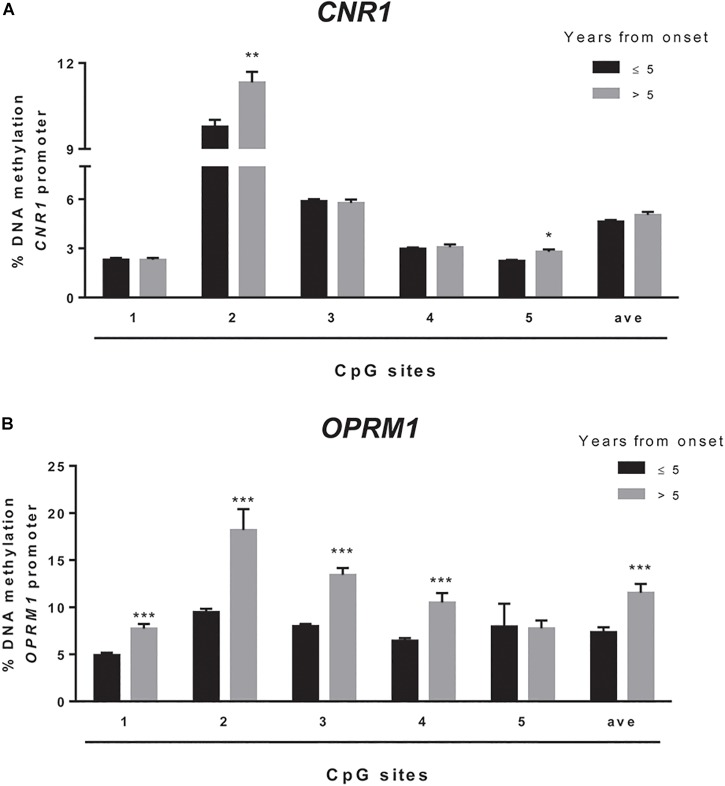
Comparison of the DNA methylation status at human *CNR1* **(A)** and *OPRM1* **(B)** promoters in the obese population stratified based on the years from obesity onset. The bars represent the mean of the % of methylation values of individual CpG sites under study as well as of the average (ave) of the CpG sites ± the SEM. Significant differences are indicated: ^∗^*P* < 0.05; ^∗∗^*P* < 0.01; ^∗∗∗^*P* < 0.001 vs. obese > 5.

We sought to extend the analysis of DNA methylation at *CNR1* and *OPRM1* promoters in the preclinical animal model to a group of humans with obesity and matched healthy controls (CTRLs). In these samples, we failed to observe any alteration of the epigenetic hallmark at both gene promoters in the overall population (Table 5). Yet, data stratification based on subject age showed significant differences at both gene promoters in individuals younger than 30 years (Figure 5). Namely, DNA methylation at *CNR1* promoter was lower in humans with obesity at CpG 5 (CTRLs = 2.62 ± 0.17, obese = 2.16 ± 0.06; *p* = 0.006), as well as at the average of the five CpG sites under study (CTRLs = 4.97 ± 0.11, obese = 5.58 ± 0.10; *p* = 0.008), when compared to age-matched controls (Figure 5A). Instead, DNA methylation at *OPRM1* promoter was significantly lower in obese subjects at CpG 1 (CTRLs = 6.57 ± 0.31, obese = 5.28 ± 0.30; *p* < 0.01), CpG 2 (CTRLs = 12.37 ± 0.44, obese = 9.85 ± 0.40; *p* < 0.001), CpG 3 (CTRLs = 11.10 ± 0.76, obese = 8.31 ± 0.27; *p* < 0.001) and CpG 4 (CTRLs = 8.22 ± 0.47, obese = 6.61 ± 0.25; *p* < 0.01), when compared to age-matched controls (Figure 5A) No differences in DNA methylation were observed between obese and healthy controls in subjects older than 30 years (Figure 5B). We also observed the DNA methylation levels on *CNR1* and *OPRM1* gene promoters in humans with obesity and Ctrl stratified for gender (Supplementary Figure S1). No differences in the % of DNA methylation of *CNR1* were observed in female and male subjects (Supplementary Figures S1A,B). Lower levels in DNA methylation at *OPMR1* were observed in human males with obesity at CpG site 3 (CTRLs = 13.45 ± 0.65, obese = 10.13 ± 0.79, *P* < 0.004), CpG site 4 (CTRLs = 9.94 ± 0.45, obese = 7.61 ± 0.52, *P* < 0.003), as well as at the average of the five CpG sites under study (CTRLs = 10.96 ± 0.49, obese = 8.72 ± 0.64, *P* < 0.010), when compared to CTRLs (Supplementary Figure S1A). No differences in the % of DNA methylation of *OPRM1* were observed in female subjects (Supplementary Figure S1B). Considering just the humans with obesity, DNA methylation differences were not observed after data stratification based on gender (Supplementary Figure S2). Focusing on the obese population only, DNA methylation differences were observed after data stratification based on the time length from obesity onset (Figure 6). Additionally, methylation at *CNR1* promoter of subjects that were obese for more than 5 years was higher than that of humans with obesity for a shorter time. In particular, we observed a significant increase at two of the five sites analyzed, namely at the second (CTRLs = 9.78 ± 0.23, obese = 11.32 ± 0.37; *p* < 0.008) and at the fifth (CTRLs = 2.23 ± 0.06, obese = 2.80 ± 0.13; *p* < 0.14) CpG site, respectively (Figure 6A). Methylation of the combined five CpG sites analyzed in the promoter region of *OPRM1* showed a significant increase in DNA methylation of humans with obesity for a long time (>5 years from onset) (CTRLs = 7.36 ± 0.51, obese = 11.51 ± 0.95; *p* < 0.001). Moreover, we observed a significant increase in the first (CTRLs = 4.91 ± 0.24, obese = 7.73 ± 0.50; *p* < 0.001), second (CTRLs = 9.49 ± 0.34, obese = 18.18 ± 2.23; *p* < 0.001), third (CTRLs = 8.00 ± 0.21, obese = 13.42 ± 0.73; *p* < 0.001), and fourth (CTRLs = 6.45 ± 0.25, obese = 10.50 ± 1.01; *p* < 0.001) CpG site analyzed (Figure 6B). Finally, differences were also observed when considering the BMI of humans with obesity, and *post hoc* group differences are reported in Supplementary Figure S3. Focusing on humans with obesity younger than 30 years, we compared the DNA methylation status at *CNR1* and *OPRM1* promoters in Preadolescents (8–12 years old), Adolescents (13–17), Young adults (18–30) (Supplementary Figure S4). A *t*-test corrected by Sidak–Bonferroni multiple comparisons indicated a significant increase of *OPRM1* methylation levels at CPG site 3 of young adults, when compared to the adolescents (Adolescent = 7.96 ± 0.26, Young adult = 9.85 ± 0.78, *P* < 0.008). Association remained for all stratified data, after Sidak–Bonferroni correction for multiple testing.

**Table 5:** DNA methylation changes at human *CNR1* and *OPRM1* gene promoters in controls (CTRL) and humans with obesity.

*Gene*	*CNR1*		*OPRM1*	
CpG sites	CTRL	Obese	CTRL	Obese
1	2.36 ± 0.14	2.27 ± 0.07	7.87 ± 0.37	7.11 ± 0.32
2	10.41 ± 0.28	10.39 ± 0.25	15.62 ± 1.20	14.76 ± 0.83
3	5.84 ± 0.24	5.72 ± 0.15	12.90 ± 0.56	12.04 ± 0.57
4	3.03 ± 0.22	2.93 ± 0.10	9.55 ± 0.38	9.03 ± 0.43
5	2.70 ± 0.13	2.52 ± 0.09	7.65 ± 0.35	8.03 ± 0.95
Average	4.87 ± 0.17	4.74 ± 0.13	10.72 ± 0.50	10.19 ± 0.47


## Discussion

The first outcome of this study is the selective up-regulation of the expression of *Cnr1*, the gene coding for CB_1_, and of *Oprm1*, the gene coding for MOP, in the hypothalamus of rats exposed to high fat diet for 5 and 21 weeks. These alterations were present at both time-points analyzed for CB_1_, that appeared to be engaged in long-lasting effects, and only at the beginning of obesity development for MOP, that appeared to be engaged in obesity onset only. Of note, we failed to observe any alteration in any other component of the ECS and of the EOS, speaking in favor of a distinct role of the two receptors in obesity.

Our data are thus consistent with many studies that already showed the hyperphagic role of CB_1_ (Williams and Kirkham, 1999; Jamshidi and Taylor, 2001; Verty et al., 2005; Koch et al., 2015) and MOP (Smith et al., 2002) in this brain region. Hypothalamic administration of the endocannabinoid anandamide (Jamshidi and Taylor, 2001) or of the cannabis extract tetrahydrocannabinol (THC) (Verty et al., 2005), both primarily acting though CB_1_ in the brain, rapidly increases food intake in rats. Studies focused on the hypothalamus documented also that high-fat feeding increases MOP protein levels in Wistar rats (Smith et al., 2002), as well as in rats susceptible to obesity (Barnes et al., 2006).

The link between these two receptors in the promotion of feeding has been proposed by Kock and colleagues, who showed that this effect can be due to CB_1_ activation on POMC neurons leading to the release of β-endorphin, an opioid neuropeptide acting on MOP (Koch et al., 2015).

Despite the great amount of research showing CB_1_ levels dysregulation centrally in animal models of obesity (South and Huang, 2008; Massa et al., 2010), there is less information about altered hypothalamic *Cnr1* mRNA levels, which anyhow have been reported by some (Kempf et al., 2007; Gamelin et al., 2016; Ramirez-Lopez et al., 2016). Consistently with our present findings, *Cnr1* mRNA levels increased in female offspring hypothalami from rat dams fed a high caloric diet (Ramirez-Lopez et al., 2016). Moreover, receptor knockdown in mice hypothalamus induced a reduction in body weight while increasing energy expenditure (Cardinal et al., 2012). On the other hand, receptor gene expression was down-regulated in the hypothalamus of rats fed with a palatable high-energy diet (Timofeeva et al., 2009). It seems noteworthy that in a recent analysis of the transcriptional regulation of ECS components in an animal model of binge eating behavior, we observed a selective epigenetic regulation of fatty acid amide hydrolase, the key enzyme for the degradation of anandamide, instead, *Cnr1* and all other ECS components were not affected (Pucci et al., 2018). This seems of particular relevance if one aims at finding specific biomarkers for different eating disorders and related disturbances (such as obesity).

The involvement of MOP gene regulation in obesity has also been investigated. MOP KO mice fed a high-fat diet were resistant to obesity (Tabarin et al., 2005), even though others also showed that MOP KO mice on a standard diet increased body weight in adulthood, when compared to wild-type littermates (Han et al., 2006). Analysis of gene expression showed increased MOP mRNA levels in hypohalami of the offspring of obese pregnant mice (Vucetic et al., 2010; Grissom et al., 2014), whereas no changes were observed in mice fed from weaning with high-fat diet for around 15 weeks (Vucetic et al., 2011).

In order to characterize MOP and CB_1_ transcriptional regulation, we sought to compare hypothalamic genes DNA methylation. We observed a consistent reduction of the epigenetic hallmark at *Cnr1* promoter, in four of the eight CpG sites under study as well as in their methylation average, but only at the earliest time-point (5 weeks). However, no changes were observed in MOP DNA methylation. These findings suggest that the temporary hypomethylation at *Cnr1* gene at the beginning of obesity development might be responsible of the early changes observed in gene expression; yet, it does not seem necessary in the long term, when chromatin might be in a poised state with mRNA levels resulting still high. At any rate, this is the first report showing DNA methylation of *Cnr1* in obesity, whereas others have already addressed MOP epigenetic regulation in mice reward-related brain regions of the offspring of pregnant dams exposed to chronic high-fat diet (Vucetic et al., 2011). In partial agreement with our data, also the latter studies failed to show alterations of methyl CpG-binding protein, as well as of histone acetylation, at MOP gene promoter in the hypothalamus (Vucetic et al., 2011). Very recently the hypothalamic increase in histone acetylation was instead reported at *Cnr1* gene promoter, and was linked to increased hypothalamic receptor expression (Almeida et al., 2019).

With the aim to extend the study of CB_1_ and MOP receptors to human obesity, we assessed whether PBMCs might mirror central nervous system defects, and we then evaluated DNA methylation at *CNR1* and *OPRM1* gene promoters in PBMCs of humans with obesity. We failed to observe any difference between controls and humans with obesity in the overall population, yet age-based stratification of the data clearly showed a significant reduction of the epigenetic hallmark at both *CNR1* and *OPRM1* promoters in younger (<30 years old) humans with obesity. As for *CNR1*, an age-dependent modulation was observed at one of the five CpG sites analyzed, as well as at their average, possibly driving an up-regulation of gene expression compared with normal weight subjects. Instead, in the same population DNA methylation was reduced in four of the five CpG sites analyzed. These differences for both receptors did not occur in subjects older than 30 years. Our findings appear of particular relevance if one attempts to identify early disease biomarkers. In agreement with this, a lower DNA methylation was also observed at genes promoters of humans with obesity with a shorter time length from disorder onset, and in those with lower BMI.

At the clinical level, it seems noteworthy that an increased *CNR1* expression in obesity has been shown at the peripheral level (Pagano et al., 2007; Sarzani et al., 2009), but never in blood samples from human subjects. Moreover, to the best of our knowledge, *OPRM1* expression in humans with obesity has not been studied yet, with the only available PET clinical study in different brain regions reporting lower MOP availability in obese subjects compared to controls (Karlsson et al., 2015).

Overall, our findings in human peripheral samples confirm the data observed centrally in the animal model, showing that regulation of *CNR1* and *OPRM1* genes is altered mainly at the early stage of phenotype development, at least in terms of the epigenetic hallmarks analyzed.

## Conclusion

In conclusion, epigenetic switching appears dynamic and temporary in the preclinical and clinical models used here to investigate *CNR1* and *OPRM1*. Alterations of DNA methylation in these two genes, observed earlier in the obese phenotype as a result of early exposure (i.e., to high-fat diet or, for instance, to stressful conditions), disappear at a later stage in life. Thus, here we clearly show the relevant role of *CNR1* and *OPRM1* transcriptional regulation as a possible biomarker for obesity and, due to the reversible nature of the epigenetic hallmark, our data open new avenue for environmental strategies of intervention. As yet, conventional environmental strategies for the treatment of obesity include lifestyle modifications of diet and physical activity, and are often insufficient (Blevins, 2010). However, this might be also due to the lack of an early diagnosis. In line with this, our data support the relevance of a preventive therapeutic approach based on environmental factors.

Of relevance, a very recent study has reported altered DNA methylation on obesity-related genes in healthy adolescents, and these alterations were suggested as early life changes possibly associated with elevated disease risk in adulthood (He et al., 2019).

### Study Limitations

We should point out that we analyzed PBMCs, composed of different cell types with different DNA methylation profiles (Bell et al., 2011). However, in order to extract homogeneous populations, cell manipulation is required, which is a laborious and difficult procedure to standardize, possibly affecting gene expression profiles (Whitney et al., 2003; Debey et al., 2004). Therefore, laser capture micro-dissected tissues might be used to address this issue in future studies. Moreover, for a better understanding of the relationship between blood and brain in terms of epigenetic modulation, it would be of interest to study also in rats transcriptional regulation of genes in blood. In addition, it seems necessary to extend the present study to a larger cohort of human subjects, possibly by using innovative methods to achieve cell-sorting, and to add different preclinical experimental models of obesity to further characterize the epigenetic regulation of *CNR1* and *OPRM1* expression, as well as the relationship between their peripheral and central modulation.

## Data Availability

The raw data supporting the conclusions of this manuscript will be made available by the authors, without undue reservation, to any qualified researcher.

## Ethics Statement

This study was carried out in accordance with the recommendations of “name of guidelines, name of committee” with written informed consent from all subjects. All subjects gave written informed consent in accordance with the Declaration of Helsinki. The protocol was approved by the Ethic Committee of IRCCS – Istituto Auxologico Italiano (RBFR12DELS_004/05C302_2013).

This study was carried out in accordance with the recommendations of Institutional Guidelines and complied with the Italian Ministry of Health and associated guidelines from European Communities Council Directive. The protocol was approved by Italian Ministry of Health no. 1610/2013.

## Author Contributions

MP, CD, CC, and MM conceived and designed the experiments. CD, MP, MM, VV, SM, EZ, and MMas performed the experiments. CD, MS, LP, MP, and CC analyzed the data. CD, CC, and AS contributed reagents, materials, and analysis tools. CD, MP, and MMac wrote the manuscript.

## Conflict of Interest Statement

The authors declare that the research was conducted in the absence of any commercial or financial relationships that could be construed as a potential conflict of interest.
