# Supplementary material for: Preclinical and Clinical Evidence for a Distinct Regulation of Mu Opioid and Type 1 Cannabinoid Receptor Genes Expression in Obesity
Source: Front Genet. 2019 Jun 14;10:523. doi: 10.3389/fgene.2019.00523 (PMC6588048; doi:10.3389/fgene.2019.00523)
Supplement: Supplementary file 1 [file Presentation_1.pdf]

Supplementary material

Table S1. List of primers used for quantitative real-time RT–PCR.

**Gene**		**5'- Forward - 3'**	**5' - Reverse - 3'**
**Endocannabinoid system**	***Cnr1***	ttccaccgtaaagacagccc	tccacatcaggcaaaaggcc
	Cnr2	ttgaccgatacctatgtctgtgc	tgctttccagaggacataccc
	Gpr55	atgctggtatgggttggaagc	atgatgcaggctctctttcg
	Trpv1	attgaacggcggaacatgacg	atctcttccagcttcagcg
	Nape-Pld	tgtcccgggttccaaagaggagc	accatcagcgtcgcgtgtcc
	Faah	atggaagtcctccaagagc	tagagctttcaggcatagcg
	Dagl	attctctccttcctcctgc	atttgggcttggtgcttcg
	Magl	atgttgaagaggctggacatgc	atgcagattccggattggc
**Opioid system**	***Oprl1***	agcttctgaagaggctgtgt	gacctcccagtatggagcag
	Oprm1	agttctgcatcccaacctcg	gcctccagattttctagctggt
	Oprd1	tcgtccggtacactaagctg	ggccacgtttccatcaggta
	Oprk1	agctcttggttccccaactg	caccacagagtagacagcgg
	Pomc	gaccaaacgggaggcgacgg	ggctctgtcgcggaaaggca
	Pnoc	tgcagcacctgaagagaatg	caacttccgggctgacttc
	Pdyn	cctgtccttgtgttccctgt	agaggcagtcagggtgagaa
	Penk	atctggctcgtagcgcttg	gtgtgcatgccaggaagttg
**Housekeeping**	***β-Actina***	agatcaagatcattgctcctcct	acgcagctcagtaacagtcc
	Gapdh	agacagccgcatcttcttgt	cttgccgtgggtagagtcat

Table S2. Details of sequences and primers employed during the analysis of DNA methylation. Bold text = CpG sites analysed; Lowercase letters = 5′ upstream sequence or intron sequence; Uppercase letters = exon region.

Gene	Primers (5' – 3')	Sequence*	CpG sites
R_Cnr1	F: GGAAGAGAGTAGGAAGATGATAG<br>Biot-R: TTCTACCAAAACTAATATACCTAACACC<br>S: AGAGAGTAGGAAGATGATAGT	…tagcttttc**cg**cctccc**cg**cccccct**cg**atactggccagtggtccccaggtgtccta**cgcg**gagtt**cg**tgttc**cg**tgttccagaagagcaaat**cg**tgctaat…	8
R_Oprm1	F: GGGAGGAGAGGAGTGGATTTT<br>Biot-R: CTTCTACCCCCCCTTACCCCACTT<br>S: AGAGGAGTGGATTTTG	…catggagac**cg**ac**cg**ac**cg**aggaggctgactctctgattgctttg**cg**caaaatccactcct…	4
H_CNR1	Included in the assay<br>PM00122031	…GACCAGGGGATGCGAAGGgtaagacctggcagagttg**cg**tttggaaatacttttc**cg**cctccc**cg**ccccct**cg**ggta**cg**accagtgtggtccccaagtgt…	5
H_OPRM1	Biot-F: GTGGGGGTAGAGTTGTTGTTTATG<br>R: TCTCTATCTCAACCAAAACTAATTTCTAT<br>S: ATCTCCATACTCCTAACTACCT	…CTGGCTACCTC**GC**ACA**GC**GGT**GC**CC**GC**CCG**GC**CTCAGTAC…	5

Open field test

Automated locomotor activity boxes (square plastic boxes measuring 43 x 43 x 30 cm, with a 25 x 3 x 25 cm virtual central zone; Med Associates, St Albans, Vermont, USA) were used to quantify spontaneous locomotor activity parameters. Activity was recorded for 10 min, starting 1 min after the placement of the animal in the test cage. Locomotor activity was recorded automatically by interruption of two orthogonal light beams (3.5 and 13 cm above the activity box floor), which were connected to automatic software. Locomotion counts were recorded when the low row of photocells was interrupted. Locomotion in the entire open field and in the central zone and entries into the central zone were computed based on interruptions of infrared light beams placed in a 2.6 x 3 x 2.6 cm orthogonal grid 3.5 cm above the box floor (Micioni Di Bonaventura et al., 2012, 2017). Increased locomotor activity in the entire field is considered a sign of behavioral arousal, and reduced locomotor activity in the central zone and numbers of entries into the central zone are considered signs of increased emotionality, anxiety or fear in mice and rats (Royce, 1977; Thiel et al., 1999; Bailey and Crawley, 2009). Between test sessions, the apparatus was cleaned with alcohol (70%) and dried with a cloth.

Table S3. Open-field test in rats with STD and rats after 5 and 21 weeks on HFD.

AFTER 5 WEEKS	ENTIRE AREA		CENTRAL AREA	
	STD	HFD	STD	HFD
**Distance travel (cm/10 min)**	3501.2 ± 319.2	3682.6 ± 361.7	95.4 ± 15.7	102.4 ± 17.8
**Vertical count (beam breaks/10 min)**	95.4 ± 13.9	92.7 ± 10.9	3.2 ± 1.2	2.5 ± 0.9
**Grooming (beam breaks/10 min)**	910.6 ± 90.6	892 ± 67.7	22.4 ± 6.3	71.5 ± 5.5
**Zone Entries (beam breaks/10 min)**			47.7 ± 6.4	52 ± 6.9
**AFTER 21 WEEKS**	**ENTIRE AREA**		**CENTRAL AREA**	
	**STD**	**HFD**	**STD**	**HFD**
**Distance travel (cm/10 min)**	3738.9 ± 330.2	2311.1 ± 203.6**	101.7 ± 6.8	43.2 ± 3.3**
**Vertical count (beam breaks/10 min)**	89.1 ± 11.6	58.2 ± 2.8*	4.0 ± 0.7	1.5 ± 0.3 **
**Grooming (beam breaks/10 min)**	939.2 ± 13.5	803.0 ± 44.2*	19.2 ± 4	6.4 ± 1.1**
**Zone Entries (beam breaks/10 min)**			56.6 ± 4.5	33.5 ± 2.6 **

Data are the mean ± S.E.M. *P <0.05, **P <0.01 vs Chow rats.

Results

Rats with standard diet and DIO rats, after 5 weeks on high-fat diet, did not differ in terms of their performance on the open field (Supplementary Table S3), traveling the same distance (cm/10 min) in the entire or in the center of the arena.

Conversely after 21 weeks on high-fat diet, the locomotor activity [$F(1,14)=13.5$; $P<0.01$] and the other behavioral parameters (vertical count [$F(1,14)=6.2$; $P<0.05$] and grooming [$F(1,14)=8.7$; $P<0.05$]) in the entire arena, were found significantly decreased in DIO rats compared to chow rats. These results were expected because of the body weight gain in DIO rats.

Interestingly DIO rats showed an anxiogenic behavior, infact the zone entries [$F(1,14)=19.5$; $P<0.01$], the distance travel in the center [$F(1,14)=58.4$; $P<0.01$] as the vertical count [$F(1,14)=10.9$; $P<0.01$] and grooming [$F(1,14)=9.5$; $P<0.01$], were significantly reduced compared to lean rats (Supplementary Table S3). These findings suggest that the chronic consumption of high energy diet increases anxiety- like behavior consistently with other works (Weber-Hamann et al., 2002; Sharma and Fulton, 2013; van Reedt Dortland et al., 2013a; van Reedt Dortland et al., 2013b).

Bailey, K.R., and Crawley, J.N. (2009). "Anxiety-Related Behaviors in Mice," in *Methods of Behavior Analysis in Neuroscience,* eds. Nd & J.J. Buccafusco. (Boca Raton (FL)

Micioni Di Bonaventura, M.V., Lutz, T.A., Romano, A., Pucci, M., Geary, N., Asarian, L., and Cifani C. (2017). Estrogenic suppression of binge-like eating elicited by cyclic food restriction and frustrative-nonreward stress in female rats. International Journal of Eating Disorders 50:624-635.

Micioni Di Bonaventura, M.V., Cifani, C., Lambertucci, C., Volpini, R., Cristalli, G., Massi, M. (2012). A2A adenosine receptor agonists reduce both high-palatability and low-palatability food intake in female rats. Behavioural Pharmacology 23:567-74.

Royce, J.R. (1977). On the construct validity of open-field measures. *Psychological Bulletin* 84**,** 1098-1106.
Sharma, S., and Fulton, S. (2013). Diet-induced obesity promotes depressive-like behaviour that is associated with neural adaptations in brain reward circuitry. *Int J Obes (Lond)* 37**,** 382-389.
Thiel, C.M., Muller, C.P., Huston, J.P., and Schwarting, R.K. (1999). High versus low reactivity to a novel environment: behavioural, pharmacological and neurochemical assessments. *Neuroscience* 93**,** 243-251.
Van Reedt Dortland, A.K., Giltay, E.J., Van Veen, T., Zitman, F.G., and Penninx, B.W. (2013a). Longitudinal relationship of depressive and anxiety symptoms with dyslipidemia and abdominal obesity. *Psychosom Med* 75**,** 83-89.
Van Reedt Dortland, A.K., Vreeburg, S.A., Giltay, E.J., Licht, C.M., Vogelzangs, N., Van Veen, T., De Geus, E.J., Penninx, B.W., and Zitman, F.G. (2013b). The impact of stress systems and lifestyle on dyslipidemia and obesity in anxiety and depression. *Psychoneuroendocrinology* 38**,** 209-218.
Weber-Hamann, B., Hentschel, F., Kniest, A., Deuschle, M., Colla, M., Lederbogen, F., and Heuser, I. (2002). Hypercortisolemic depression is associated with increased intra-abdominal fat. *Psychosom Med* 64**,** 274-277.

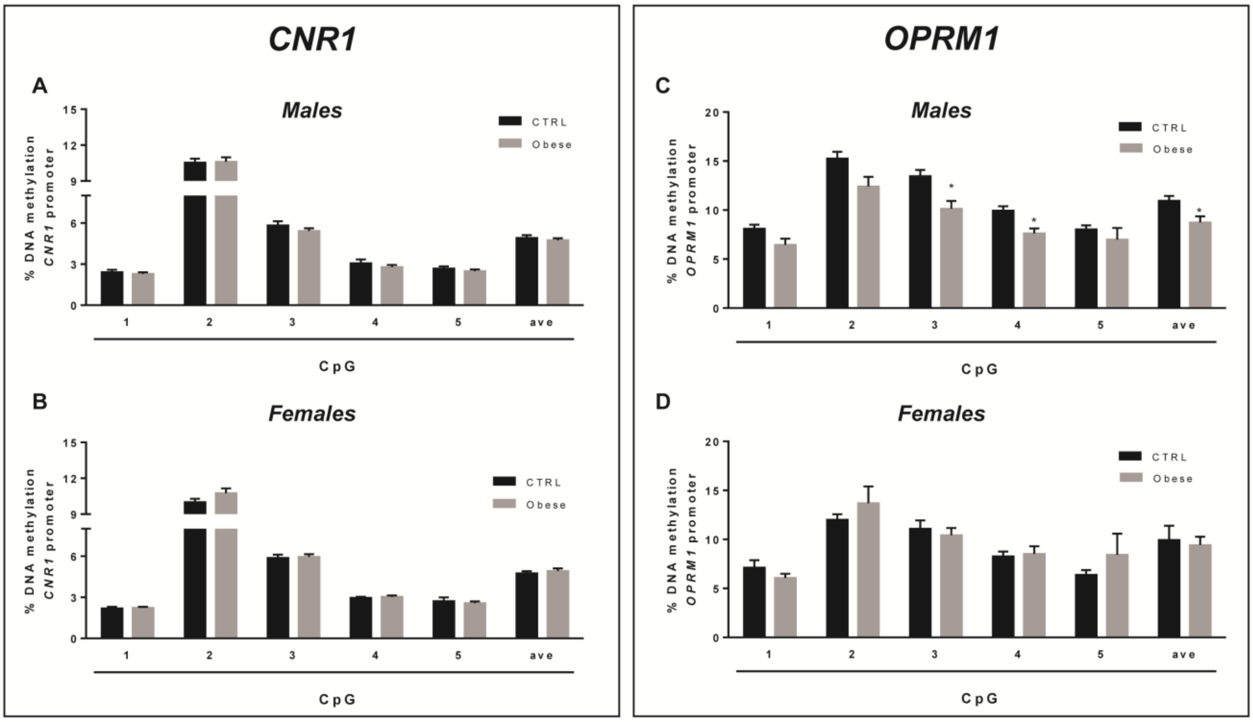

Figure S1: Comparison of the DNA methylation status at the human *CNR1* (A and B) and *OPRM1* (C and D) promoters in the human with obesity and control (CTRL) subjects stratified based on gender (Males = A and C; Females = B and D). The bars represent the mean of the % of methylation values of individual CpG sites under study as well as of the average (ave) of the CpG sites ± the SEM. Significant differences are indicated: *P < 0.05 vs CTRL.

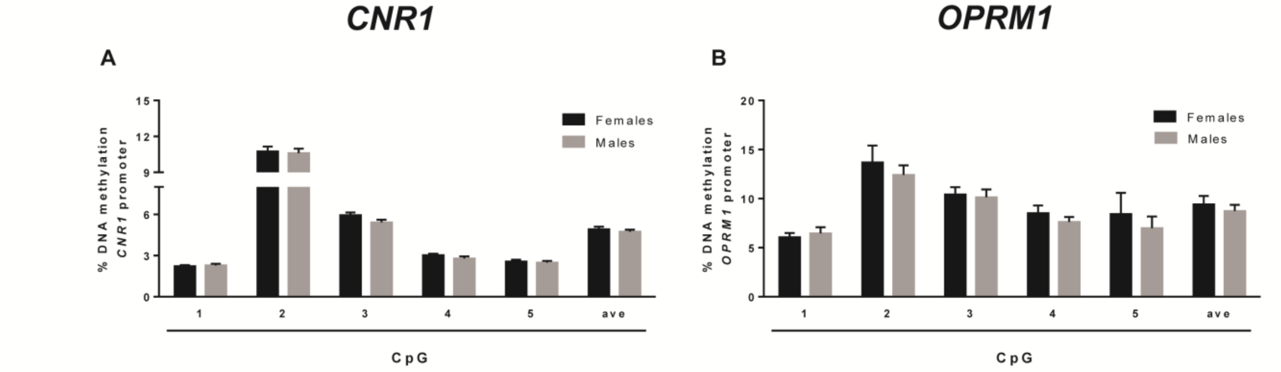

Figure S2: Comparison of the DNA methylation status at the human *CNR1* (A) and *OPRM1* (B) promoters in the human with obesity stratified based on gender. The bars represent the mean of the % of methylation values of individual CpG sites under study as well as of the average (ave) of the CpG sites ± the SEM.

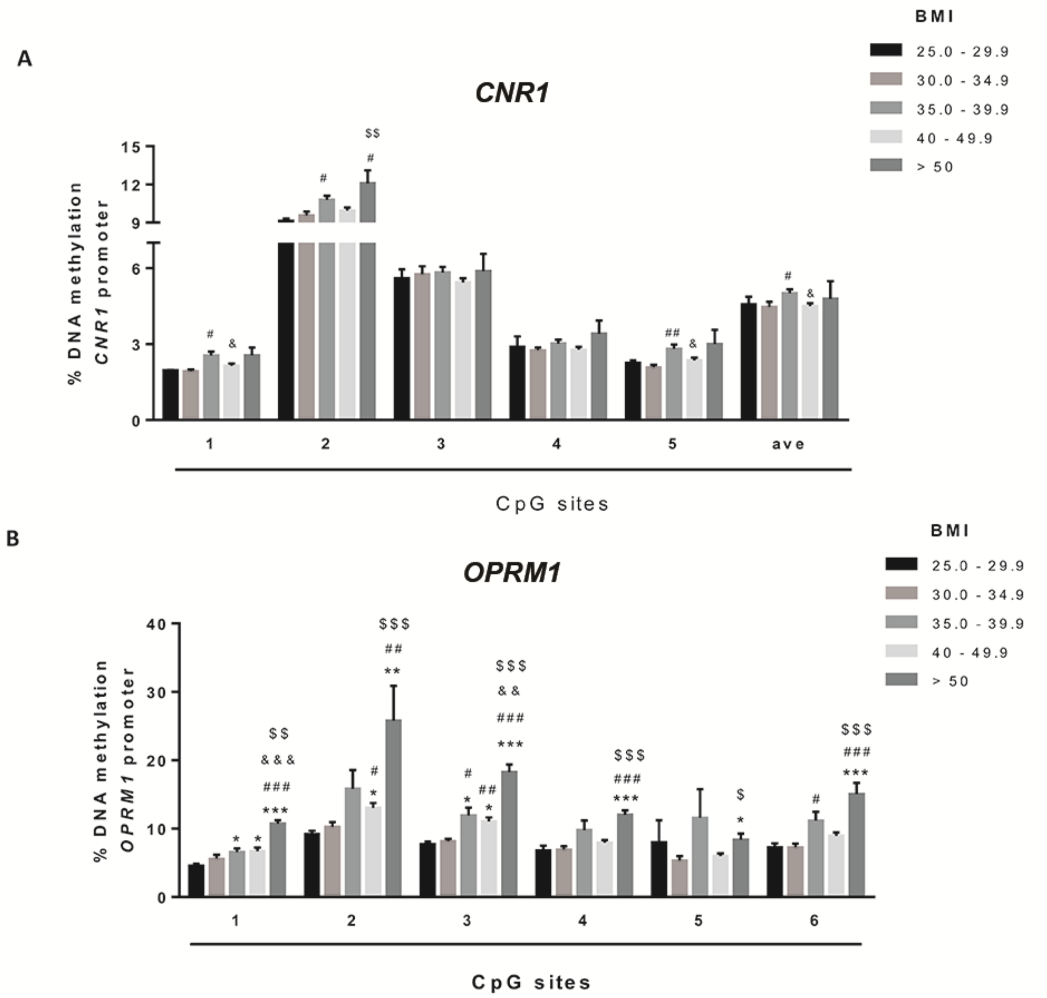

Figure S3. Comparison of DNA methylation status at human *CNR1* (A) and *OPRM1* (B) promoters in the obese population stratified based on body mass index (BMI). The bars represent the mean of the % of methylation values of individual CpG sites under study as well as of the average (ave) of the CpG sites ± the S.E.M. Significant differences are indicated: *, **,*** P < 0.05, 0.01, 0.001 vs 25.0 - 29.9, $^{\#, \#\#, \#\#\#}$P < 0.05, 0.01, 0.001vs 30.0 - 34.9; $^{\&, \&\&, \&\&\&}$ P < 0.05, 0.01, 0.001 vs 35.0 - 39.9; $^{\$, \$\$, \$\$\$}$ P < 0.05, 0.01, 0.001 vs 40.0 - 49.9.

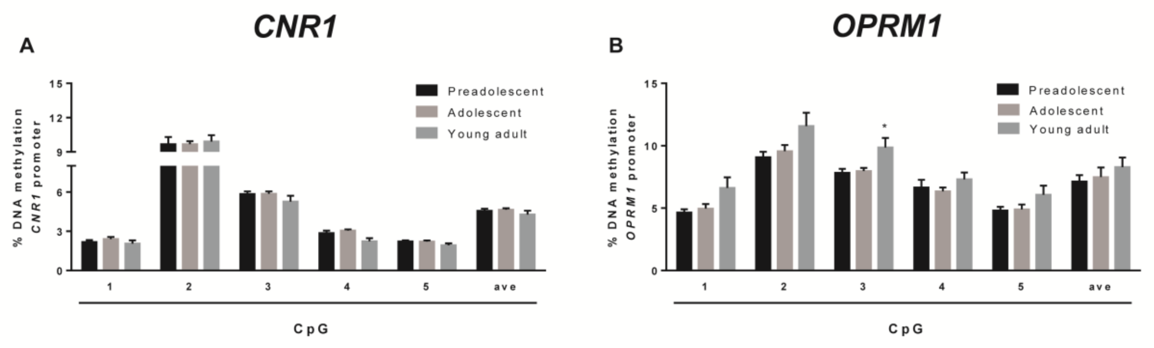

Figure S4: Comparison of the DNA methylation status at the human *CNR1* (A) and *OPRM1* (B) promoters in the human with obesity (> 30 years old) stratified based on age: Preadolescent (8-12 years old), Adolescent (13-17), Young adult (18-30). The bars represent the mean of the % of methylation values of individual CpG sites under study as well as of the average (ave) of the CpG sites ± the SEM. *P < 0.05 vs Adolescent.
